# Blood pressure trajectories after stroke: Do they matter?

**DOI:** 10.1111/jch.14330

**Published:** 2021-07-23

**Authors:** Simona Lattanzi, Afshin A. Divani, Mauro Silvestrini

**Affiliations:** ^1^ Neurological Clinic Department of Experimental and Clinical Medicine Marche Polytechnic University Ancona Italy; ^2^ Department of Neurology University of New Mexico, Albuquerque New Mexico USA

In the current issue of *The Journal of Clinical Hypertension*, the study by Dr. Fan and colleagues aimed to explore longitudinal blood pressure (BP) trajectory patterns and determine their prognostic role in stroke outcomes.^1^ By group‐based trajectory modelling, 353 patients with acute ischemic stroke were categorized into five systolic and four diastolic BP subgroups based on BP values recorded during the first 24 h after treatment.^1^ Groups were classified according to BP levels (ie, low, medium, and high) and BP changes (ie, slow decline, rapid decline, and persistent fluctuation). Compared to the stable‐moderate BP trajectory, the continuous fluctuation, very high‐level systolic BP (SBP) group had a significantly increased risk of early neurological deterioration, and the rapid drop‐high SBP and rapid drop‐high level diastolic BP patterns were inversely associated with early neurological improvement.^1^ A U‐shaped correlation was found between SBP trajectories and 90‐day functional outcome, being slow drop‐low SBP and fluctuating‐very high SBP trajectories associated with a higher risk of unfavorable outcome. The continuous fluctuation, very high diastolic BP group was also linked with an increased likelihood of a 3‐month poor recovery.^1^

The results of this study provide useful insights to advance our understanding of the intriguing relationship between BP variations after stroke and clinical outcomes. Advances in stroke treatment have reduced the overall mortality rate, whereas the number of survivors with complications and disability has increased.^2^, ^3^, ^4^ Thereby, there is the need to identify the factors that may influence stroke patients’ clinical course.

## 1 BLOOD PRESSURE TRAJECTORIES AFTER STROKE: EVIDENCE FROM CLINICAL STUDIES

Most studies on the effects of BP during acute and subacute stroke phases on clinical outcome have used one time‐point BP values and only a few studies have focused on BP trajectory patterns.

The effects of BP changes on outcome in acute ischemic stroke and the impact of baseline values and history of hypertension have been explored in 791 patients registered in the Acute Stroke Registry and Analysis of Lausanne.^5^ The outcome measure was the functional status at 3 months after stroke. An optimal outcome was identified in patients with a range of acute SBP of 110–180 mm Hg and SBP reduction. The optimal magnitude of BP reduction was proportional to acute SBP levels according to an oblique descending channel: a reduction of SBP by 10–20% was associated with optimal outcome in the range of acute SBP of 120–140 mm Hg, and a reduction of SBP greater than 20% predicted a further improvement in the 140–170 mm Hg acute SBP range.^5^ Increase and magnitude of increase in BP were associated with worse outcome irrespective of initial BP, except in patients with initially low values in whom BP modification did not significantly affect prognosis. The optimal outcome in normotensive patients showed a similar oblique descending channel. This channel was, however, shifted upwards suggesting that patients with an initially low BP could not tolerate BP reduction but benefit from mild‐to‐moderate BP increase.^5^ Hypertensive patients could tolerate elevated acute SBP better than normotensive patients, and extreme values of SBP and changes did not have a striking impact on the outcome.^5^

Among 316 patients included in the Blood Pressure and Clinical Outcome in TIA or Ischemic Stroke Study who presented with ischemic stroke and admission SBP ≥160 mm Hg, the analysis of BP values during the first 7 days after onset identified three SBP trajectories: sustained high SBP (T1), moderate decrease (T2), and rapid decrease in SBP (T3).^6^ The incidence of poor outcome was 25.9%, 13.5%, and 9.8% in T1, T2, and T3 groups, respectively. Compared with T1 group, the decrease in SBP in T2 and T3 groups was associated with a significantly lower risk of unfavorable 3‐month functional outcome.^6^

The Scandinavian Candesartan Acute Stroke Trial was a multicenter, randomized controlled, double‐blind trial of candesartan in acute stroke, which recruited 2029 patients presenting within 30 h of acute stroke and with SBP ≥140 mm Hg.^7^ According to change in BP between baseline and day 2, patients were divided into groups with increase/no change, small decrease (0–14 mm Hg), moderate decrease (14–28 mm Hg), or large decrease (≥28 mm Hg) in SBP. Patients with a large decrease or increase/no change in SBP had a significantly increased risk of early adverse events defined as recurrent stroke, stroke progression, and symptomatic hypotension during the first 7 days relative to patients with a small decrease. Patients with an increase/no change in SBP had a significantly increased risk of poor neurological status at 7 days compared with the other groups; no differences were observed across groups in long‐term functional outcome at 6 months.^7^

Five SBP trajectories of high, high‐to‐moderate‐low, moderate‐high, moderate‐low, and low were defined in 4036 patients with acute ischemic stroke and elevated BP from the China Antihypertensive Trial in Acute Ischemic Stroke trial who had their BP measured from day 1 until hospital discharge or death.^8^ Patients with the highest SBP, averaging 176 mm Hg during the first 24 h and 167 mm Hg during days 2–7 had the highest odds for major disability, death, recurrent stroke, and cardiovascular disease. Patients who experienced a rapid BP reduction from approximately 180 mm Hg to < 140 mm Hg during the first 3 days had reduced risks of poor clinical outcomes compared with patients without rapid reduction. Furthermore, patients with moderate‐low SBP, averaging 139 mm Hg during days 2–7 had the lowest risk of adverse clinical outcomes.^8^

The influence of BP trajectory on subsequent clinical events up to 1 year after stroke was explored in 8376 patients with cerebral infarct admitted within 24 h of symptom onset and included in a prospective multicenter registry.^9^ Modeling of BP during the first 24 h after admission classified patients into five trajectory groups: low, moderate, rapidly stabilized, acutely elevated, and persistently high SBP. Compared with the moderate SBP group, the acutely elevated and persistently high SBP groups had significantly increased risk of having vascular events, including recurrent stroke, all‐cause death, or a composite of recurrent stroke, myocardial infarction, and all‐cause death.^9^

Interestingly, the magnitude of BP reduction was a predictor of in‐hospital outcomes also in patients with hemorrhagic stroke. In a cohort of 757 patients with spontaneous intracerebral hemorrhage, unsupervised functional principal components analysis revealed that mean SBP over 24 h and SBP reduction within the first 6 h from stroke accounted for 76.8% of the variation in SBP trajectories.^10^ An increase in SBP reduction was significantly associated with poor outcome at discharge. Compared with SBP reduction < 20 mm Hg, worse outcomes were observed for 40–60 mm Hg and > 60 mm Hg reduction in BP. Furthermore, the relationship between BP decrease and outcome varied according to initial hematoma volume: a smaller reduction in SBP was associated with good outcome in small and medium‐size hematomas and with decreasing probability of severe outcome in patients with large hematomas.^10^

## 2 BLOOD PRESSURE CHANGES AFTER STROKE: FROM PATHOPHYSIOLOGY TO CLINICAL CARE

Although the management of BP in acute stroke has been investigated in several trials, optimal BP levels have not been well‐established yet, and uncertainty remains about whether, when, and how BP should be lowered.^11^, ^12^

During the acute phase of stroke, cerebral blood flow follows the systemic arterial pressure due to impairment of autoregulatory mechanisms, where both extremely high and low BP values have been associated with poor outcomes. High BP may increase the risk of hemorrhagic transformation of infarcted area, favor hematoma expansion by promoting ongoing bleeding and re‐bleeding and enhance brain formation. Of note, the rise in BP may also favor recanalization and represent a protective response to guarantee perfusion in the presence of dysfunctional cerebral autoregulation and raised intracranial pressure.^13^, ^14^ Low BP may enhance cerebral damage by decreasing blood flow to potentially viable penumbra and perihematomal region, and increase the risk of tissue ischemia and lesion size expansion.^15^, ^16^ Extreme BP fluctuations can also contribute to the breakdown of the blood‐brain barrier and increase intracranial pressure.

Importantly, BP dynamics may serve as an important modifiable and prognostic factor. The relationship between BP and outcome is not simply linear, and BP changes during the acute stroke phase can play a role in the development of complications.^17^, ^18^ In this regard, group‐based trajectory modelling of BP is receiving attention as an emerging approach to account for the dynamic nature of BP over time, which cannot be summarized into a single mean value or variability index. In patients with stroke, BP may reasonably be grouped by distinct trajectories, which are characterized by distinctive clinical features, risk for unfavorable recovery, and subsequent vascular events. In particular, currently available evidence suggests how not only the initial BP values, but also the direction and magnitude of associated changes over the first hours are associated with stroke outcome, and both large reductions and increases in BP may have detrimental effects.

Additional research is still warranted to explore the relevance of BP trajectories in different stroke populations, assess the reciprocal interaction of causes and effects of BP variations, and investigate the utility of trajectory patterns in identifying the best candidates for BP‐lowering therapy. Indeed, it is likely that a variety of patient‐specific factors such as age, stroke type, recanalization treatment, infarct volume, status of collaterals, cardiovascular comorbidities, and prior antihypertensive therapy may influence the range of optimal BP values and vulnerability to the detrimental effects of BP changes.^5^, ^19^ It also seems rationale that there is no one‐size‐fits‐all approach and the optimal therapeutic approach to stroke should be based on multivariable models and strategies tailored to individual patient characteristics.^20^, ^21^, ^22^, ^23^ The increasing availability of computational resources to analyze big data may expedite the development of integrated and personalized models of stroke care.

## CONFLICT OF INTEREST

None.
